# A Fluorescence-Coupled Assay for Gamma Aminobutyric Acid (GABA) Reveals Metabolic Stress-Induced Modulation of GABA Content in Neuroendocrine Cancer

**DOI:** 10.1371/journal.pone.0088667

**Published:** 2014-02-13

**Authors:** Joseph E. Ippolito, David Piwnica-Worms

**Affiliations:** 1 Mallinckrodt Institute of Radiology, Washington University School of Medicine, St. Louis, Missouri, United States of America; 2 BRIGHT Institute, Washington University School of Medicine, St. Louis, Missouri, United States of America; 3 Department of Genetics, Washington University School of Medicine, St. Louis, Missouri, United States of America; 4 Department of Cancer Systems Imaging, University of Texas M.D. Anderson Cancer Center, Houston, Texas, United States of America; H. Lee Moffitt Cancer Center & Research Institute, United States of America

## Abstract

Pathways involved in the synthesis of the neurotransmitter gamma-aminobutyric acid (GABA) have been implicated in the pathogenesis of high grade neuroendocrine (NE) neoplasms as well as neoplasms from a non-NE lineage. Using The Cancer Genome Atlas, overexpression of the GABA synthetic enzyme, glutamate decarboxylase 1 (*GAD1*), was found to be associated with decreased disease free-survival in prostate adenocarcinoma and decreased overall survival in clear cell renal cell carcinomas. Furthermore, *GAD1* was found to be expressed in castrate-resistant prostate cancer cell lines, but not androgen-responsive cell lines. Using a novel fluorescence-coupled enzymatic microplate assay for GABA mediated through reduction of resazurin in a prostate neuroendocrine carcinoma (PNEC) cell line, acid microenvironment-induced stress increased GABA levels while alkaline microenvironment-induced stress decreased GABA through modulation of GAD1 and glutamine synthetase (GLUL) activities. Moreover, glutamine but not glucose deprivation decreased GABA through modulation of GLUL. Consistent with evidence in prokaryotic and eukaryotic organisms that GABA synthesis mediated through GAD1 may play a crucial role in surviving stress, GABA may be an important mediator of stress survival in neoplasms. These findings identify GABA synthesis and metabolism as a potentially important pathway for regulating cancer cell stress response as well as a potential target for therapeutic strategies.

## Introduction

The neuroendocrine (NE) system is a diffuse network of cells distributed throughout various tissues and organs that can produce local or systemic effects on physiology through the secretion of hormones or neurotransmitters. Although some cells arise from neural crest, the majority are derived from multipotential epithelial progenitor cells [1]. Neuroendocrine (NE) carcinomas, believed to arise from the NE system, occur in virtually all anatomic locations and display a wide spectrum of phenotypic behaviors from benign to metastatic [2], [3]. For example, “classic” carcinoid tumors or low-grade NE carcinomas are well-differentiated, have a low mitotic index, and resemble NE cell hyperplasia [2], [3], while small cell carcinomas or high grade NE carcinomas are aggressive and poorly differentiated [4]–[9]. High grade NE carcinomas characteristically have numerous areas of necrosis, a high mitotic index [2], [3], and a poor prognosis. Conventional therapies do not improve patient survival in patients with high grade NE carcinoma [10].

Adenocarcinomas, which are much more common, arise from an epithelial origin and display a glandular growth pattern [11]. Interestingly, adenocarcinomas can exhibit NE features characterized molecularly as the expression of gene products associated with the NE cell lineage. This phenomenon has been demonstrated in many types of adenocarcinoma, including those from lung, prostate, and colon, and correlates with tumor aggressiveness, metastasis, and shortened survival [12]–[19]. Specifically, in the case of prostate cancer, the expression of NE features positively correlates with metastatic potential and castration-resistant growth [19]–[21]. Unfortunately, the biology of NE cells as well as their contributions to organ homeostasis remain incompletely defined, in part due to the paucity of these cells in normal organs [22].

Previously, we used a combination of gene expression profiling, mass spectrometry, and nuclear magnetic resonance spectroscopy to elucidate a series of metabolic networks implicated in aggressive NE cancers using a genetically-engineered mouse model of metastatic prostate NE carcinoma, and a derivative Prostate NE Carcinoma (PNEC) cell line, wherein the cryptdin 2 promoter drives expression of oncogenic large T antigen (CR2-TAg) [13], [23]–[25]. Results of these analyses identified the synthesis and metabolism of gamma-aminobutyric acid (GABA) derived from both tricarboxylic acid (TCA) cycle intermediates and polyamines (collectively referred to as the GABA shunt) as a metabolic network prominently up regulated in aggressive NE carcinomas [13].

Modern specialized instrumentation used for metabolic analyses, such as mass spectrometry (MS) and nuclear magnetic resonance (NMR) are expensive, require significant training to operate, and may not be readily available to many academic laboratories. Classical enzymatic-based metabolite quantitation, on the other hand, provides a cost-effective, readily accessible, sensitive means for quantitative analysis of metabolites [26]. Although unable to provide simultaneous information on the quantities of multiple metabolites, enzymatic quantitation offers the ability to simultaneously quantitate a given metabolite across many samples on a high-throughput platform [27]–[29].

Pyridine nucleotide-based enzymatic assays that couple the production of NADH or NADPH (collectively referred to as NAD(P)H) to a biochemical reaction are attractive, as these reduced nucleotides fluoresce and can therefore be used for quantitative readout of enzyme activity or metabolite levels. However, background fluorescence in biological tissues can limit the sensitivity of NAD(P)H detection [26].

Multiple strategies to enhance the photonic output and improve the sensitivity of enzymatic reactions have been developed [26], [30]–[32]. One strategy involves the coupling of NAD(P)H synthesis to mitochondrial lipoyl dehydrogenase (diaphorase) which activates chemical substrates for chromogenic or fluorescence assays. Resazurin is one such compound that can be used for enzymatically-coupled and cell viability assays [27], [33], [34]. Here, we report a novel enzymatic assay for the quantitation of GABA and successfully use this assay to characterize changes in cellular GABA in PNEC cells under metabolic stress.

## Materials and Methods

### Reagents

All chemical and enzymatic reagents were purchased from Sigma.

### Cell Culture

All cell lines routinely tested mycoplasma negative. All cell lines were cultured at low passage under conventional conditions according to ATCC protocols in a 95% O_2_/5% CO_2_ incubator at 37°C. A self-attaching subclone of the Prostate Neuroendocrine Cancer (PNEC) cell line [35] was cultured as previously described [36]. Briefly, DMEM/F12 medium (Invitrogen) was supplemented with 10% heat inactivated FBS (Gibco), 1% non-essential amino acids (NEAA, Gibco), B27 serum supplement (Invitrogen), 5 ng/mL EGF (BDBiosciences), 5 ng/ml bFGF (Sigma), and 15 mM 4-(2-hydroxyethyl)-1-piperazineethanesulfonic acid (HEPES) buffer (Gibco). All cells were grown to a maximum of 75% confluency. Cells were trypsinized, neutralized with growth media and passaged. Cell pellets for RNA or biochemical assays were spun down at 125×g for 5 min at 4°C. Cells were washed with ice cold phosphate buffered saline, and centrifuged again. Supernatants were aspirated and pellets snap frozen in liquid nitrogen. All samples were stored at −80°C until further use.

### Evaluation of Survival Curves from The Cancer Genome Atlas (TCGA)

The cBioPortal for Cancer Genomics [37], [38] provided through The Cancer Genome Atlas Data Portal was used to access and visualize clinical and gene expression datasets of human prostate adenocarcinoma [39] and human clear cell renal cell carcinoma [40]. Only the “mRNA expression z-scores vs. Normals” option was checked. “All tumors” were selected for the query. RNASeq data was used for the clear cell carcinoma datasets. Survival curves were assessed based upon expression of genes within the GABA shunt. Samples exhibiting increased *GAD1* expression (z score>+2 or +3) and decreased *GLUL* expression (z<–2) were identified and clinical information exported. Survival curves were constructed with GraphPad Prism.

### Real Time PCR Primer Design

Primers for real time PCR were designed using PrimerBLAST. *GAD1* (NM_000817.2), but not 18S rRNA (*RNA18S5*;NR_003286.2), was designed across an intron-exon junction. Selected primers did not have any predicted off-target amplification in the human genome. Primer melting temperatures were designed between 61°C and 63°C. Primers were tested with PCR from cDNA synthesized from NCI-H660 and LNCaP cell lines. Amplicons of expected length (GAD1∶145 bp; 18S: 182 bp) were obtained from cDNA preparations and were not evident in PCR reactions using genomic DNA as a template or water only. Melt curves and amplification efficiencies of all primer pairs were performed. The primer sequences are as follows: *GAD1* (forward): 5′-ATGGGGTTCGCACAGGTCATCC-3′, *GAD1* (reverse): 5′-TCCATGAGGACAAACACTGGTGCA-3′; *RNA18S5* (forward): 5′-GGGCATTCGTATTGCGCCGC-3′, *RNA18S5* (reverse): 5′- GAATAACGCCGCCGCATCGC-3′.

### Real Time PCR

RNA was isolated from snap frozen cell pellets using the RNeasy kit (Qiagen). Following RNA extraction, all RNA samples were aliquoted and stored at −80°C. All RNA samples were treated with TURBO Dnase (Ambion) for removal of genomic DNA. All RNA used for quantitative PCR was immediately reverse transcribed with the iScript kit (BioRad). The purity of the cDNA was assessed with 18S rRNA PCR amplification of the cDNA sample and a negative control sample of RNA that lacked the reverse transcriptase. All samples used for subsequent QPCR experiments did not have detectable genomic DNA contamination, as evidenced by a lack of an 18S amplicon following 40 cycles of the reaction lacking reverse transcriptase. Each sample was tested in triplicate at 50 ng cDNA. Real-time PCR was performed with SYBR green master mix (BioRad) and a 100 nM concentration of the specific primer set in a Bio-Rad CFX96 PCR instrument (95°C for 10 min, then 40 cycles of 95°C for 30 s, 61°C for 1 min, and 74°C for 1 min). 18S rRNA primers were used as an internal standard.

### Metabolic Stress Experiments

For nutrient deprivation experiments, DMEM/F12 media lacking glucose, pyruvate, and glutamine (USBiological) was used. Media was supplemented with the following components: heat inactivated dialyzed serum with a 10 KDa molecular weight cutoff (Sigma), 1% non-essential amino acids (NEAA, Gibco), 5 ng/ml EGF (BDBiosciences), and 5 ng/ml bFGF (Sigma). Glucose and glutamine (Gibco) were supplemented to the specialized media when needed. Glucose was added to the medium using standard DMEM/F12 formulation concentrations of 17.5 mM. Glutamine was used at 6 mM. Prior to metabolic stress experiments, PNEC cells were plated at a density of 400,000 cells per well in a 12 well plate with 1.5 mL of standard growth media. Cells were allowed to grow for 24 hours. Media was then exchanged for 1.5 mL stress media. For all pH stress experiments, conventional PNEC growth media was used without bicarbonate or HEPES buffers and was modified as follows. For acid stress, 2-(*N*-morpholino)ethanesulfonic acid (MES), pH 6.5 was added for a final concentration of 20 mM. For conventional pH, HEPES, pH 7.4 was added for a final concentration of 20 mM and sodium bicarbonate added for a final concentration of 0.34 g/L. For alkaline stress, tris(hydroxymethyl)aminomethane (Tris) pH 8.5 was added for a final concentration of 20 mM and sodium bicarbonate added for a final concentration of 0.34 g/L. Cells were then incubated in a humidified chamber without CO_2_ at 37°C for 24 hours.

### Sample Preparation

Sample preparation for the enzymatic assay was modified from previously described methods [13], [26]. For stress experiments, media was quickly aspirated from the wells and cells were rinsed with 1 mL PBS. For acid and base stress experiments, wells were rinsed with saline buffered to the appropriate pH with 20 mM MES or HEPES as above. Cells were then lysed in 200 µL ice-cold lysis solution (50 mM sodium hydroxide and 1 mM EDTA) and gently shaken for 30 sec. An equal volume of 100 mM hydrochloric acid was then added to acidify the lysate. Plates were covered with adhesive PCR film seal and incubated in a water bath at 60°C for 30 minutes to destroy endogenous enzyme activity as well as endogenous NADH. Following incubation, plates were briefly centrifuged at 100×g (Allegra 6R; Beckman-Coulter) to eliminate condensate. The film was removed and 100 µL 400 mM Tris Base was added to each well, for a total volume of 500 µL lysate in each well (final pH approximately 8). Lysates were centrifuged at 15,000×g for 5 minutes to pellet debris and supernatants were collected and frozen at −80°C until further use.

### Resazurin-based Assay for GABA and Succinic Semialdehyde Determination

Following reagent optimization, the mastermix for quantifying GABA and succinic semialdehyde (SSAL) in samples consisted of 0.063 U/mL GABase, 0.063 U/mL diaphorase, 6.25 µM resazurin, 100 µM nicotinamide adenine dinucleotide phosphate (NADP), 5 mM alpha-ketoglutarate, and 3.125 µM dithiothreitol (DTT) in 100 mM Tris base, pH 8.8. The mastermix was made fresh prior to each experiment.

A mastermix for quantifying only SSAL included the above reagents as well as 50 mM 2-aminoethyl hydrogen sulfate, an inhibitor of the GABA transaminase component of the GABase enzyme preparation. The inhibitor was added to the mastermix for 10 minutes at room temperature prior to the addition of mastermix to the samples. A fresh preparation of the inhibitor was prepared prior to all experiments.

Aliquots of sample (10 µL) were added to wells of a 96-well clear bottom black culture plate (Costar) followed by addition of 90 µL of mastermix with an electronic multichannel pipette. Two aliquots of the same sample were used for determination of total GABA plus SSAL or SSAL alone on the same plate. Unless otherwise specified, the reaction was conducted at room temperature and was protected from light for 30 minutes. The signal obtained from resorufin, the fluorescent product of resazurin, was quantitated using a FLUOstar Optima microplate reader (BMG Labtech) with a 544 nm excitation/590 nm emission filter set (gain, 1327; 10 flashes per well). Kinetic analyses were performed with 1 minute cycles.

The signal for total GABA plus SSAL in each sample was subtracted from the SSAL alone signal to obtain the signal for GABA. Standard curves for GABA and SSAL were constructed for quantitation and were corrected for the reaction blank with and without the presence of 2-aminoethyl hydrogen sulfate. GABA and SSAL measurements were normalized to protein in the lysates, quantitated with the bicinchoninic acid (BCA) assay (Pierce) using a bovine serum albumin standard curve. Data was processed with GraphPad Prism. Nutrient and pH stress groups were analyzed with one way ANOVA and Dunnett post-test for statistical analysis.

### Resazurin-based Assay for Glutamate Determination

An enzymatic assay for the determination of glutamate was performed as described previously [41]. Briefly, the reaction components included 10 µM resazurin, 4 U/mL glutamate dehydrogenase, 0.25 U/mL glutamic-pyruvic transaminase, 100 µM alanine, 0.5 mg/mL NAD+, and 0.5 U/mL diaphorase in 100 mM Tris-HCl, pH 7.5. 10 µL of the above samples were added to 90 µL of the reaction mix and assays conducted in the dark at room temperature. Resorufin signal was measured at 15 minutes. Nutrient and pH stress groups were analyzed with one way ANOVA and Dunnett post-test for statistical analysis.

### NAD(P)H Fluorescence-based GABase Assay

The assay was modified from Passoneau [26]. The mastermix consisted of 0.08 U/mL GABase, 5 mM alpha-ketoglutarate, 500 µM NADP, 100 µM DTT and 4 mM EGTA in 100 mM sodium pyrophosphate, pH 8.6. Aliquots of sample (10 µL) were added to 96-well optical plates (Falcon 353261) followed by 90 µL of mastermix. The reaction was conducted at room temperature for 1 hour and measured in a FLUOstar Optima with a 340 nm excitation/450 nm emission filter set (gain, 2103, 10 flashes per well).

### Western Blotting of Metabolic Enzymes

PNEC cells were plated, stressed, and washed as described above. 100 µl of ice-cold RIPA buffer (10 mM Tris pH 8.0, 1 mM EDTA, 0.5 mM EGTA, 140 mM sodium chloride, 0.1% sodium dodecylsulfate (SDS), 0.1% sodium deoxycholate, and 1% Triton X-100) containing 1 mM sodium orthovanadate and phenylmethylsulfonylfluoride (PMSF) was added. Cells were scraped from the wells on ice and added to microfuge tubes. Samples were centrifuged for 10 minutes at 10,000×g at 4°C to remove insoluble precipitate. Protein was quantitated with the BCA assay as described above.

A total of 30 µg protein was loaded onto a 4–12% Bis-Tris polyacrylamide gel (Bolt; Life Technologies) with reducing agent per manufacturer specifications. The gel was transferred to an immobilon membrane (Millipore) and blocked with phosphate buffered saline containing 5% bovine serum albumin and 0.1% tween–20. The following primary antibodies were used: anti-GAD1 (MAB5406; Millipore) at 1∶5000 dilution, anti-ALDH5A1 (HPA029716; Sigma) at 1∶1000 dilution, anti-GLUL (MAB302; Millipore) at 1∶1000 dilution, and anti-ACTB (ab8226; Abcam) at 1∶4000 dilution. Primary antibodies were incubated overnight at 4°C. Secondary goat anti-mouse or anti-rabbit horseradish-peroxidase conjugated antibody (Cell Signaling) was then used at 1∶5000 dilution. The same blot was used to examine expression of all proteins. Following use of each antibody, the blot was stripped with Western Re-probe (G Biosciences) according to manufacturer specifications. Blots were developed and imaged using the WesternBright Quantum detection kit (Advansta).

### Enzymatic Assays

PNEC cells were plated, stressed, and washed as described above. Cells were scraped in 100 µl of ice-cold lysis buffer (100 mM sodium phosphate, pH 7.0, 20 µM pyridoxal phosphate, and 0.1% triton X-100) and repeated a second time for each well. Samples were briefly sonicated (2–3 seconds) on ice and subsequently centrifuged for 10 minutes at 10,000×g at 4°C to remove insoluble material. Prior to sample quantitation, all assays were evaluated for linearity with respect to time and amount of added lysate. All enzyme activities were normalized to time and the amount of protein in the lysate. Statistical analyses were conducted as described above.

#### Glutamate decarboxylase assay

The method was modified from an original protocol [42]. The assay buffer contained 100 mM sodium phosphate pH 7.0, 250 µM pyridoxal phosphate, 50 mM glutamate, and 0.4% beta-mercaptoethanol. A reaction blank was conducted for each sample and did not contain pyridoxal phosphate or glutamate. 50 µL of lysate was added to 50 µl of reaction buffer in a PCR plate and incubated for up to 8 hours at 37°C in a thermocycler without heated lid. The assay was terminated with the addition of 17 µL of 350 mM HCl and heated for 30 minutes at 60°C in a thermocycler. The plate was removed and briefly centrifuged to eliminate condensate. 17 µL of 400 mM Tris base was added to neutralize the sample and the plate centrifuged for 10 minutes at 1000×g to eliminate protein precipitate. Wells were then diluted tenfold for measurement of GABA, the product of the enzymatic reaction, using the GABase-resazurin assay as described above.

#### Glutamine synthetase assay

The method was modified from an original protocol using spectrophotometric determination of γ-glutamyl hydroxamate [43]. The assay buffer contained 50 mM imidazole buffer, pH 6.8, 50 mM hydroxylamine, pH 6.8, 100 mM glutamine, 25 mM sodium arsenate, 0.2 mM adenosine diphosphate (ADP), and 0.5 mM manganese chloride. A reaction blank was conducted for each sample and did not contain arsenate, ADP, or glutamine. 10 µL of lysate was added to 90 µl of reaction buffer in a PCR plate and incubated for up to 8 hours at 37°C in a thermocycler without heated lid. The reaction was terminated with the addition of 100 µL of developing reagent (0.37 M iron (III) chloride, 0.3 M trichloroacetic acid, 0.6 M HCl). Samples were centrifuged to remove precipitate and absorbance measured at 505 nm. Sample absorbance was calibrated with γ-glutamyl hydroxamate standard.

#### ALDH5A1 (SSADH) assay

The method was modified from previous protocols [26], [44]. The assay buffer contained 100 mM Tris pH 8.8, 50 mM potassium chloride, 1 mM dithiothreitol, 2.5 mM nicotinamide adenine dinucleotide (NAD), 1.25 mM SSAL, and 0.02% bovine serum albumin. A reaction blank was conducted for each sample and did not contain SSAL. 10 µL of lysate was added to 90 µl of reaction buffer in a PCR plate and incubated for up to 8 hours at 45°C in a thermocycler without heated lid. Reactions were transferred to a microplate and the absorbance of NADH, the enzymatic product, was measured at 355 nm. Sample absorbance was calibrated with NADH standard.

## Results

### Clinical Implications of the GABA Shunt

**Figure 1:**
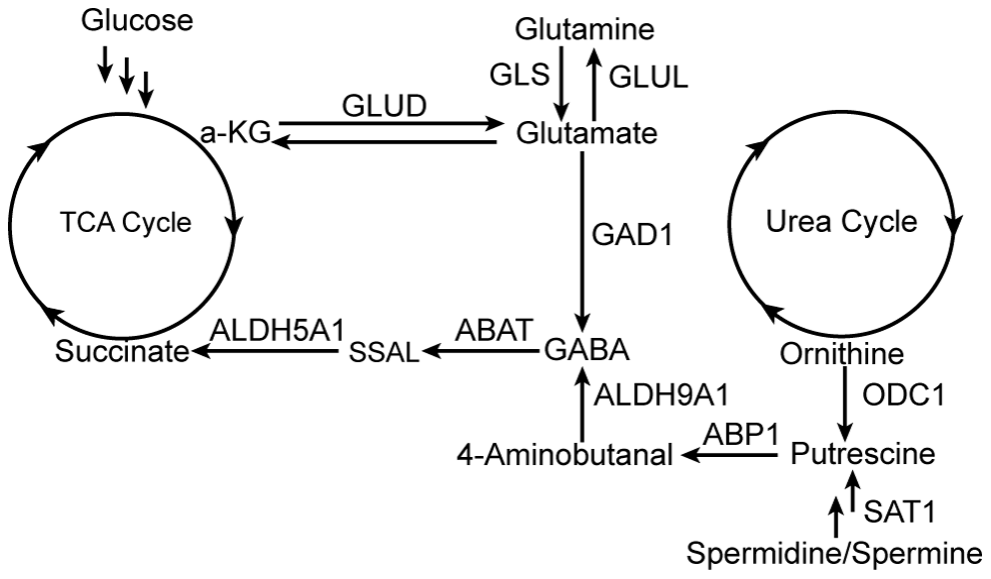
GABA synthetic and metabolic pathways in NE cells. GLUD: glutamate dehydrogenase; GLS: glutaminase; GLUL: glutamate-ammonia ligase; GAD1: glutamate decarboxylase; ODC1: ornithine decarboxylase; ABP1: diamine oxidase; ABAT: GABA transaminase; SSAL: succinic semialdehyde; ALDH5A1: succinic acid semialdehyde dehydrogenase; ALDH9A1: aldehyde dehydrogenase; SAT1: spermidine/spermine N-acetyltransferase.

We have previously determined with expression analyses that GABA synthesis is enriched in high grade NE malignancies [13] and validated the presence of a functional GABA shunt in the prostate NE cancer (PNEC) cell line using a combination of gene expression profiling, metabolite and enzyme activity quantitation [13], [25]. GABA can be derived from tricarboxylic acid (TCA) cycle intermediates and polyamine/cationic amino acid products that couple GABA metabolism to multiple pathways in cellular energy metabolism (**Fig. 1**). In one pathway, GABA is produced by glutamate decarboxylase 1 (GAD1)-mediated decarboxylation of glutamate derived from either glutamine or alpha-ketoglutarate from the TCA cycle. In a second pathway, products of polyamine and cationic amino acid degradation through putrescine undergo redox-mediated conversion to GABA. GABA can then be metabolized by aminobutyrate transaminase (ABAT, GABA-T) and succinic acid semialdehyde dehydrogenase (ALDH5A1, SSADH) into succinate for entry into the TCA cycle.

**Figure 2:**
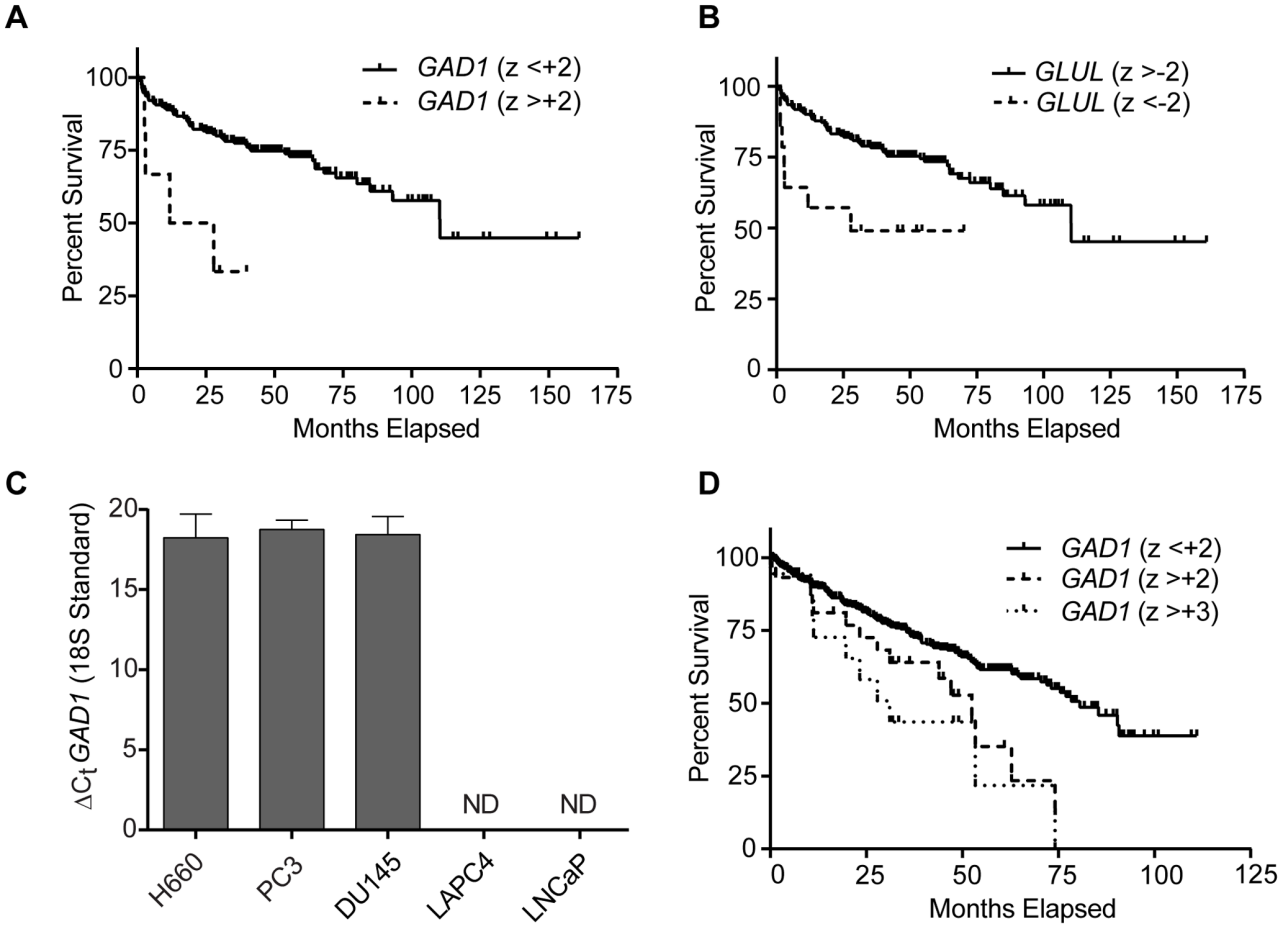
GABA shunt gene expression in human cancers. **A.** *GAD1* mRNA overexpression in prostate adenocarcinomas correlates with decreased disease-free survival. Tumors with a *GAD1* expression z-score greater than +2 display decreased disease-free survival (median 19.8 months) relative to tumors that do not have increased *GAD1* expression (median 110.3 months; p = 0.002). **B.** *GLUL* downregulation in prostate adenocarcinomas correlates with disease-free survival (median 27.9 months) relative to tumors without *GLUL* downregulation (median 110.3 months; p = 0.006). **C.** *GAD1* mRNA expression in prostate cancer cell lines measured with quantitative PCR. *GAD1* expression is detectable in castrate-resistant cell lines NCI-H660, PC3, and DU145, but not androgen-responsive cell lines LNCaP and LAPC4. 18S rRNA was used as an internal standard. ΔC_t_ is the difference of the threshold cycle of *GAD1* and the threshold cycle of 18S rRNA. ND: Not detected. **D.** *GAD1* mRNA overexpression in clear cell renal cell carcinomas correlates with reduced overall survival. Tumors with a *GAD1* z-score >+2 display decreased overall survival (median 52.5 months) relative to tumors that do not have increased *GAD1* expression (median 80.6 months; p = 0.01). Tumors with a *GAD1* z-score >+3 display a greater decreased median survival time (median 31.1 months) relative to the remainder of the tumors (median 78.4 months; p = 0.002).

First, to determine if overexpression of GABA shunt enzymes were detectable in human prostatic malignancies and if overexpression correlated with adverse clinical events, we used the cBioPortal [37], [38] of TCGA to interrogate a publicly-available prostate cancer gene expression dataset and its corresponding clinical information [39] for cohorts of patients whose cancer overexpressed components of the GABA shunt. Out of all of the enzymes listed in Fig. 1, *GAD1* was the only gene whose overexpression was associated with reduced disease-free survival. We identified a total of 6 patients out of a total of 216 patients (2.8%) whose cancers displayed *GAD1* overexpression with a z-score greater than +2. Patients with cancers that overexpressed *GAD1* had a median disease free survival time of 19.8 months versus 110.3 months (p = 0.003) (**Fig. 2A**). This cohort of patients had a wide range of prostate serum antigen (PSA) levels ranging from 3.5 to 40.2 mg/dL, Gleason grades ranging from 3+3 to 4+5, and staging ranging from T2C to T3B. Although there was only one documented case of metastatic lymphadenopathy at the time of surgery, all samples demonstrated extracapsular extension. Interestingly, decreased expression of glutamine synthetase (*GLUL*) that can potentially compete with the GAD1 enzyme for glutamate was also associated with decreased disease free survival. A total of 14 patients with z-scores less than –2 were identified with a median survival of 27.9 months versus 110.3 months (p = 0.006; **Fig. 2B**).

As the expression of NE genes has been implicated in castrate-resistant prostate cancer, specifically with respect to the increased expression of the NE marker aromatic amino acid decarboxylase (*DDC*) in castrate-resistant prostate cancer [21], we determined if *GAD1* mRNA was enriched in human castrate-resistant prostate cancer. Interestingly, *GAD1* mRNA expression was detectable only in castrate-resistant cell lines (**Fig. 2C**), with similar expression levels in the NCI-H660 NE cancer cell line and the PC3 and DU145 adenocarcinoma cell lines. No detectable expression of GAD1 was identified in either of the androgen-responsive LNCaP and LAPC4 cell lines.

Because of a report of GAD1 protein expression in a subset of renal cell carcinomas [45], we interrogated a recently published study of the clear cell subtype of renal cell carcinomas [40] provided through the cBioPortal to determine if there were similar survival characteristics. We identified 39 patients out of a total of 499 patients (8%) whose tumors overexpressed *GAD1* mRNA with a z score greater than +2. This cohort displayed a significantly decreased overall survival relative to the total clear cell cancer population with a median survival time of 52.5 months compared to 80.6 months (p = 0.01). We then isolated a cohort of *GAD1* overexpressing tumors (n = 18) with a z-score greater than +3, and identified a further decrease in overall survival with a median survival time of 31.1 months compared to 78.4 months (p = 0.002) (**Fig. 2D**). There was no significant effect of *GLUL* mRNA expression on patient survival in this population.

### Assay Development

**Figure 3:**
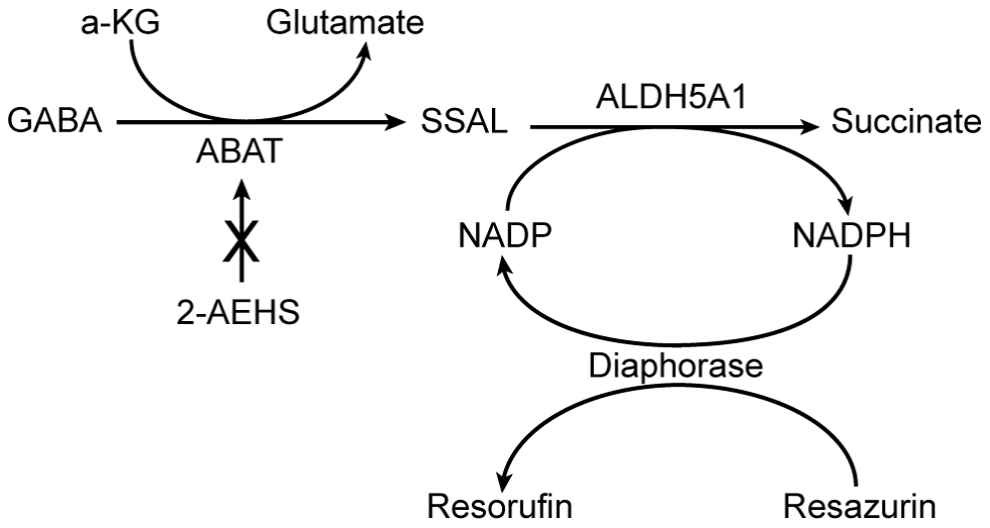
Schematic of the fluorescence-coupled GABA assay. The GABase preparation, a combination of ABAT and ALDH5A1 enzymes from *P. fluorescens* converts GABA to succinate through the conversion of alpha-ketoglutarate to glutamate and NADP to NADPH. Although NAD or NADP can potentially be used as substrates by ALDH5A1, NADP is conventionally used as a cofactor for this preparation. Succinic semialdehyde (SSAL), an intermediate of GABA metabolism is also metabolized by the GABase preparation. Diaphorase oxidizes the NADPH and reduces resazurin to fluorescent resorufin. Application of 2-aminoethyl hydrogen sulfate (2-AEHS), an inhibitor of ABAT can be used to determine the contribution of SSAL to the total signal generated from GABA and SSAL in cell lysates.

The potential for GABA to have prognostic significance in identifying subsets of cancer patients with decreased survival prompted us to develop an assay for GABA that could be widely adopted and independent of technically challenging instrumentation such as mass spectrometry and NMR. The schematic of the coupled reaction required to quantitate GABA is provided in **Fig. 3**. The commercially available “GABase” preparation from *Pseudomonas fluorescens* is a combination of ABAT and ALDH5A1 enzyme activities that oxidize GABA through a series of two reactions into succinate, resulting in reduction of the cofactor NADP to NADPH. NADPH is then oxidized to NADP by mitochondrial diaphorase thus coupling the reduction of resazurin into fluorescent product resorufin. Although ALDH5A1 can use both NAD+ and NADP as substrates, NADP has conventionally been used as a cofactor with this enzyme preparation [26].

### Assay Optimization

**Figure 4:**
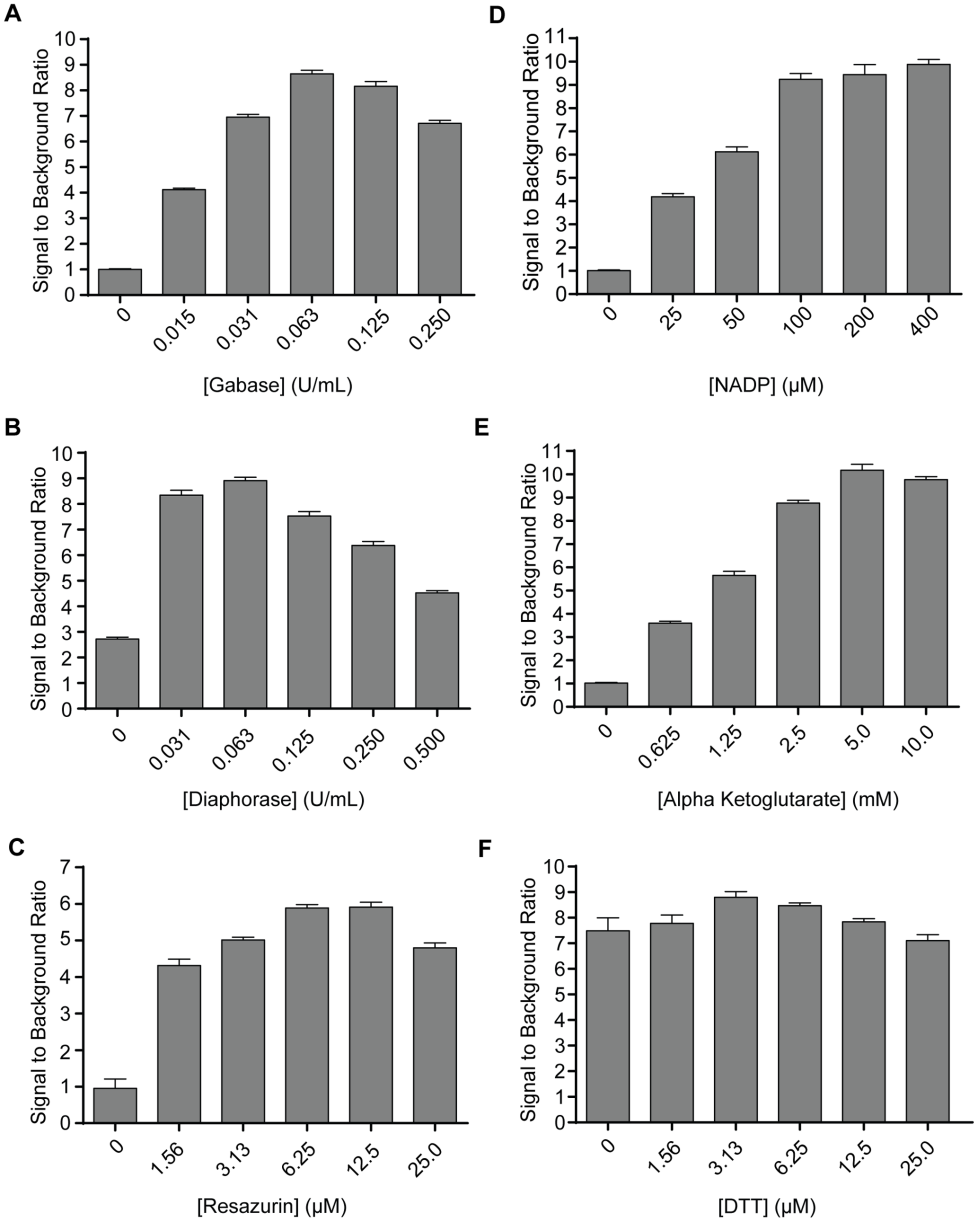
Optimization curves for GABase-diaphorase-resazurin coupled assay. Each component of the reaction mix (**A–F**) was titrated to determine the optimal concentration to achieve the highest signal to background ratio. The final reaction mix based upon these curves was 6.25 µM resazurin, 0.063 U/mL GABase, 0.063 U/mL diaphorase, 100 µM NADP, 5 mM alpha-ketoglutarate, and 3.125 µM DTT. Data were obtained in triplicate.

A classical NADPH fluorescence enzyme activity assay [26] was used as a foundation for optimization of individual components of a resazurin-based readout of GABA. Each component was titrated over a range of concentrations, keeping all other components constant (**Fig. 4**). As expected, the assay was sensitive to resazurin, demonstrating a 5.9-fold increase in signal over background at a concentration of 6.25 µM. As for the enzymatic components, there was an 8.6-fold increase in signal relative to background with 0.063 units/mL of GABase, and an 8.9-fold increase in signal over background with 0.063 units/mL of diaphorase enzyme preparations. Interestingly, although removal of GABase resulted in complete loss of signal over background, removal of diaphorase enzyme from the reaction mix resulted in a signal still 2.7-fold above background. Upon further analysis, it was found that the commercial GABase preparation contained NAD(P)H-dependent resazurin-reducing activity in the absence of GABA, which would explain this finding (data not shown).

The importance of enzymatic cofactors was also demonstrated (**Fig. 4**). NADP, a cofactor for the ALDH5A1 component of the GABase preparation, added at 100 µM concentration, demonstrated a significantly increased signal of 9.2-fold relative to background. Similarly, alpha-ketoglutarate, a cofactor for the aminobutyrate transaminase component of GABase, demonstrated 10.1-fold signal to background at a concentration of 5 mM. Removal of either of these cofactors from the reaction resulted in complete loss of signal over background.

Dithiothreitol (DTT), a reducing agent used to enhance the activity of GABase in standard NADPH fluorescence assays [26], had only a weak effect in the coupled assay. Signal over background in the absence of DTT was 7.5-fold compared to a maximum of 8.8-fold signal over background at a concentration of 3.13 µM. Reaction component concentrations with the highest signal over background were selected for use in the optimized resazurin assay composition for all future experiments (**Fig. 4**).

### Deconvolution of the Signal Obtained from GABA and Succinic Semialdehyde

Although the GABase strategy has been used in the direct enzymatic determination of GABA in biological samples, the mechanism by which this assay works can also detect succinic semialdehyde in the sample due to the presence of two enzymatic activities in the GABase preparation: aminobutyraldehyde transaminase (ABAT) that converts GABA to succinic semialdehyde (SSAL) and ALDH5A1 that converts SSAL to succinate, producing NADPH. Therefore, the signal generated from this reaction mix can in principle arise from a combination of GABA and SSAL in samples.

**Figure 5:**
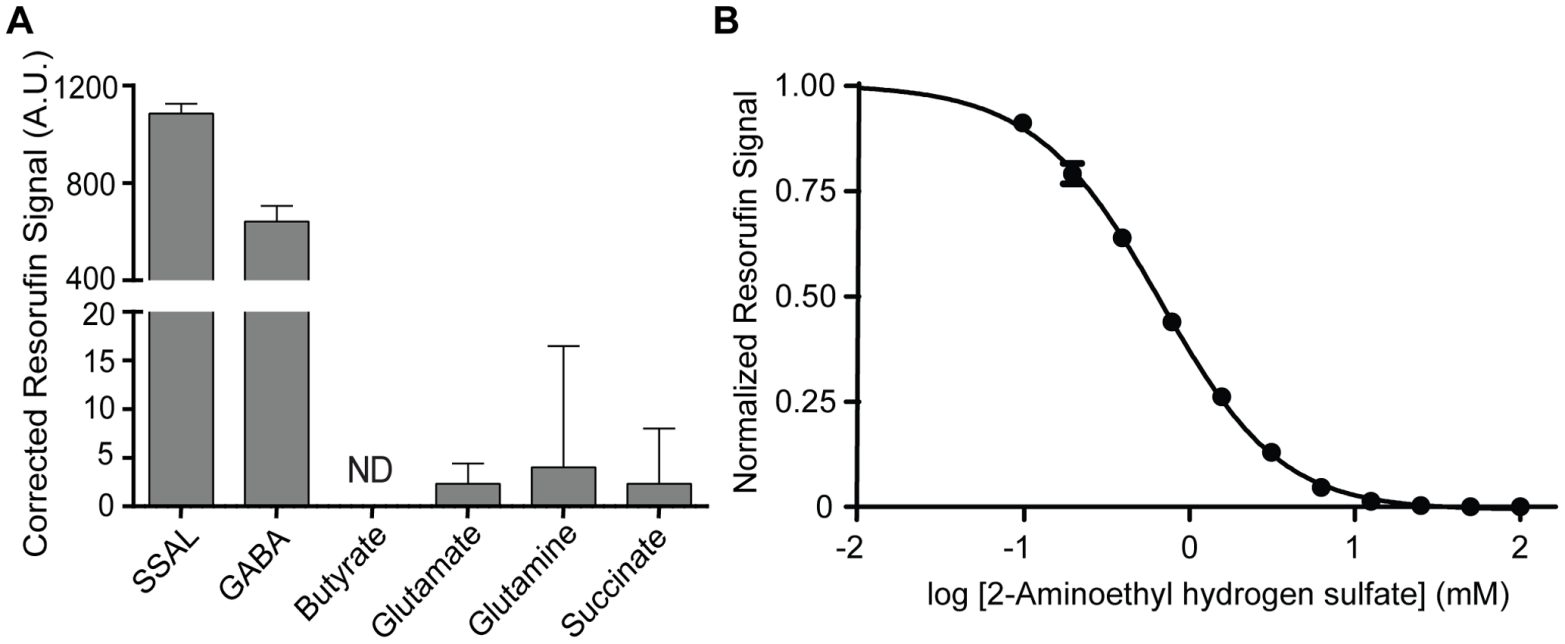
Specificity of the GABase assay. **A.** Metabolic standards (25 µM) were added to reaction mix and kinetically observed for 30 minutes. When compared to metabolites similar in chemical structure, GABA and SSAL are the only compounds that generate measurable signal in the coupled assay. Note break in scale to visualize residual activities. ND: No signal detected over background. Fluorescence signal is measured in arbitrary units (A.U.) and is corrected against the sample blank. **B.** Inhibition curve of 2-aminoethyl hydrogen sulfate, an inhibitor of the GABA transaminase (ABAT) component of the GABase preparation. At concentrations equal to or greater than 25 mM, the resorufin signal from GABA is completely abolished, allowing for the NADPH (hence resorufin signal) produced by succinic aldehyde dehydrogenase (ALDH5A1) component of the GABase preparation to be specific for succinic semialdehyde (SSAL). Data were obtained in triplicate and normalized to signal in the absence of inhibitor.

We demonstrated this experimentally, by determining the signal generated from pathway metabolites similar in structure to GABA including SSAL, glutamate, glutamine, succinate, and butyrate. At 25 µM, GABA and SSAL were the only compounds that demonstrated signal significantly different from background, indicating that the resazurin-coupled assay was specific to GABA and SSAL (**Fig. 5A**).

To circumvent this potential dual-specificity problem, we used an inexpensive inhibitor of ABAT, 2-aminoethyl hydrogen sulfate (ethanolamine-O-sulfate) [46], [47]. Addition of this inhibitor to the mastermix preparation would therefore inhibit ABAT activity in the GABase preparation, allowing for specific quantitation of SSAL in the sample. Furthermore, subtraction of signal obtained in the presence of inhibitor from signal obtained without inhibitor would result in GABA-specific signal. This methodology has previously been applied to an enzymatic assay for GABA and SSAL that uses the less sensitive NAD(P)H fluorescence method [48].

As expected, we observed a concentration-dependent effect of 2-aminoethyl hydrogen sulfate on signal obtained from 100 µM GABA (**Fig. 5B**). Measurable GABA signal was observed up to 25 mM. For this reason, we used 2-aminoethyl hydrogen sulfate at a concentration of 50 mM when required to confer specificity in subsequent experiments.

### Mechanics of the Coupled Assay

**Figure 6:**
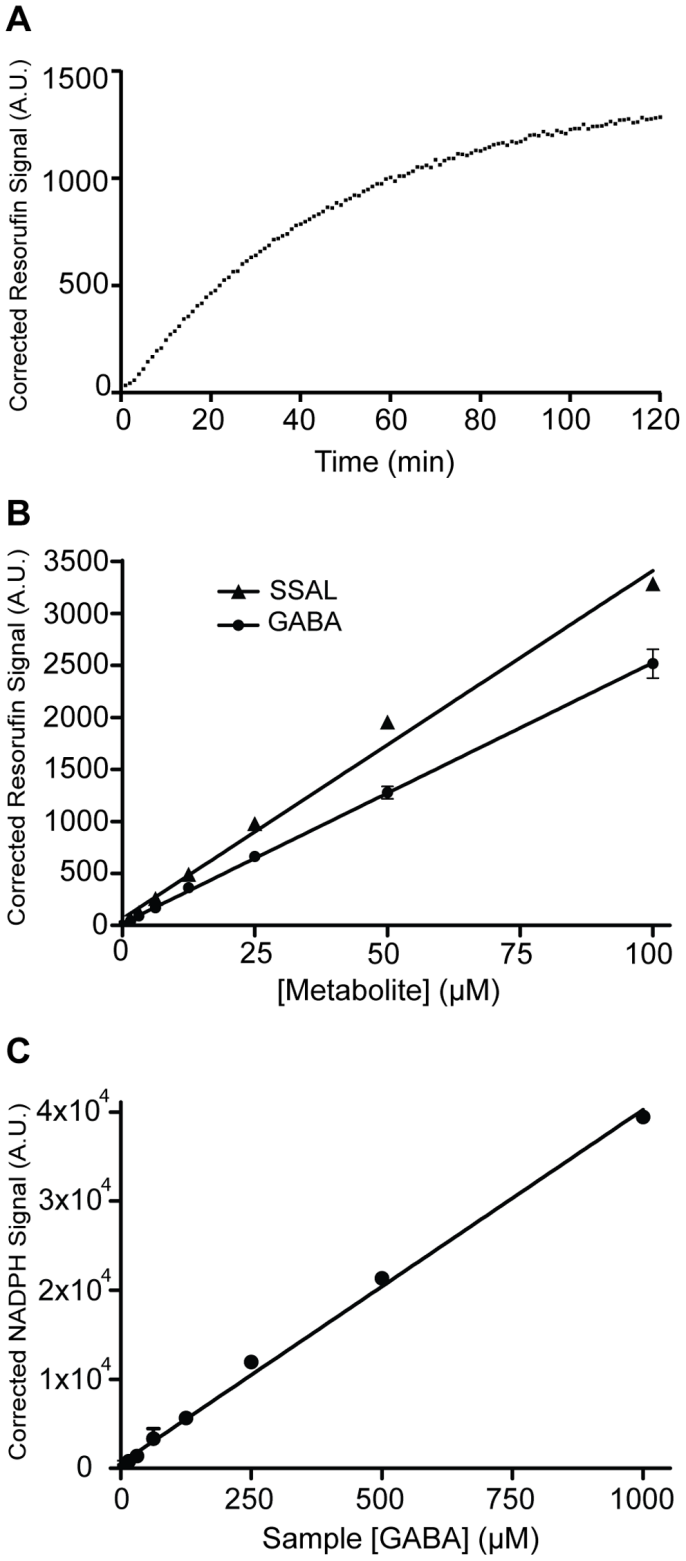
Mechanics of the GABase assay. **A.** Representative kinetics for the coupled GABase assay using 25 µM GABA. Plateau kinetics are seen after 30 minutes. The kinetic curve represents an average of triplicate measurements. **B.** Linearity of the assay. GABA and SSAL standards (10 µL) at the concentrations indicated were added to reaction mix (90 µL) and measured after 30 minutes. The limit of detection for the assay was 0.78 µM GABA and 0.41 µM SSAL. The limit of quantitation was 2.6 µM GABA and 1.4 µM SSAL. The Z’ factor for the assay was 0.8 for GABA and 0.9 for SSAL. **C.** Standard curve of [GABA] using a conventional NADPH fluorescence assay. This assay demonstrated lower sensitivity than the coupled resazurin assay when carried out under optimum conditions in an optical plate. The limit of detection for the assay was 67 µM. The limit of quantitation for the assay was 223 µM. The Z’ factor for the assay was 0.9. Fluorescence signal is measured in arbitrary units (A.U.) and is corrected against the sample blank. Data were obtained in triplicate.

The kinetics of resorufin formation were analyzed in the presence of 25 µM GABA and 25 µM SSAL (**Fig. 6A**). The formation of resorufin was linear with time through 30 minutes followed by asymptotic behavior through 120 minutes. Therefore, a 30 minute time point was selected to generate maximal signal within the region of linearity for all additional experiments. GABA or SSAL standards (10 µL) were added to reaction mix (90 µL). Signal generated from resorufin was linear to 100 µM GABA or SSAL with an R^2^ value of 0.997 for GABA and 0.992 for SSAL (**Fig. 6B**). Calculation of the “Z factor”, a measure of assay robustness [49], revealed a value of 0.8 for GABA and 0.9 for SSAL, indicating a robust assay for both metabolites.

The limit of quantitation (LOQ) and the limit of detection (LOD) were calculated as described previously [50]. The limit of detection is defined as 3σ/S and limit of quantitation is defined as 10σ/S where σ is the standard deviation of the background and S is the slope of the standard curve. Using these equations as applied to our coupled assay, the limit of detection of GABA and SSAL was 0.78 µM and 0.41 µM, respectively, and the limit of quantitation was 2.6 µM and 1.4 µM, respectively. For reference, a NADPH fluorescence assay for GABA was much less sensitive, revealing a limit of detection of 67 µM and limit of quantitation of 223 µM (**Fig. 6C**).

### Sample Preparation Optimization

We sought to develop an efficient sample preparation protocol that efficiently lysed tissue, could be conducted in a microplate format, did not destroy GABA and SSAL, and was compatible with the enzymatic assay. We modified a previously published protocol [25], [26] that used sodium hydroxide to lyse tissue, followed by the addition of excess hydrochloric acid, and heating at 60°C for 30 minutes to destroy endogenous NAD(P)H and enzyme activity that would interfere with the assay, followed by neutralization with Tris base.

**Figure 7:**
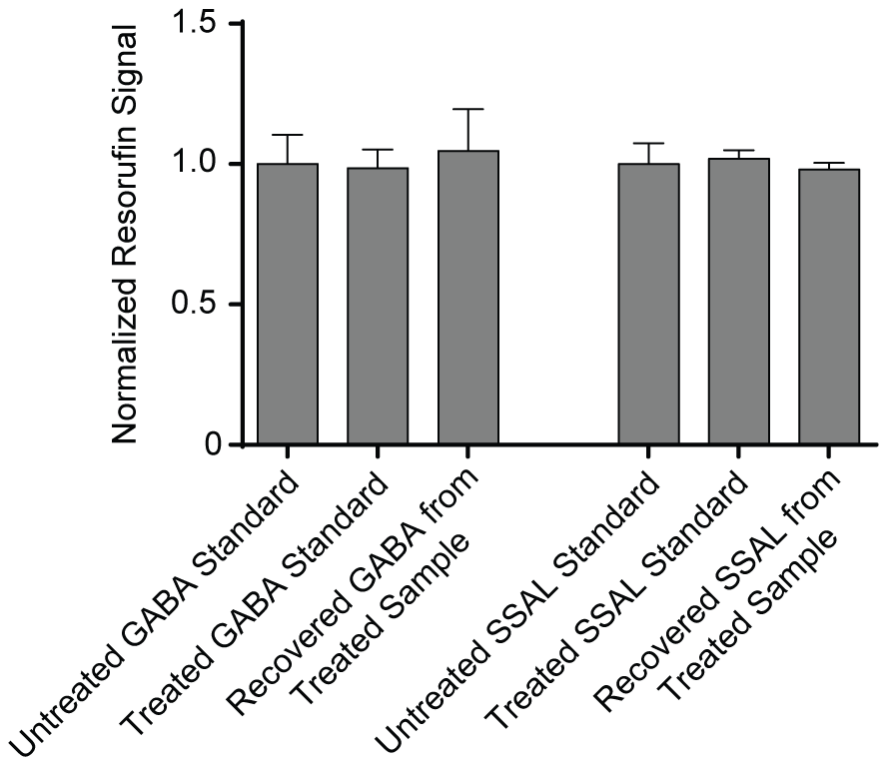
Effects of sample preparation on GABA and SSAL stability and recovery. 25 µM of GABA or SSAL standard was treated with the sample preparation protocol consisting of 50 mM sodium hydroxide followed by heating at 60°C for 40 minutes in excess 50 mM HCl and then neutralization with 400 mM Tris base. There was no significant difference in signal obtained from a 25 µM GABA or SSAL standard versus 25 µM GABA or SSAL treated with the sample preparation technique. To test recovery of GABA and SSAL from tissue, GABA or SSAL standard was added to the LNCaP prostate cancer cell sample at a final concentration of 25 µM and subjected to the sample preparation protocol. The signal recovered from the sample was subtracted from the signal obtained from an identical tissue sample without added standard, demonstrating quantitative recovery of GABA and SSAL from tissues using this technique. Fluorescence signal was measured and all data normalized to untreated standards. Data were obtained in triplicate.

To determine if this sample preparation method was compatible with GABA and SSAL quantitation, we applied a known concentration of GABA or SSAL (25 µM final concentration) to an aliquot of lysed LNCaP cells, a human prostate adenocarcinoma cell line that contains low GABA shunt activity [25]. Using lysed LNCaP cells without exogenously applied GABA or SSAL as comparators, the difference in GABA signal obtained from the two LNCaP sample groups was compared to 25 µM GABA or SSAL standards prepared in water and 25 µM GABA or SSAL standards subjected to the sample preparation method. We quantitatively recovered the GABA and SSAL standards from the tissue lysate and demonstrated that GABA and SSAL were unaffected by the sample preparation method. (**Fig. 7**).

### Assay Limitations

**Figure 8:**
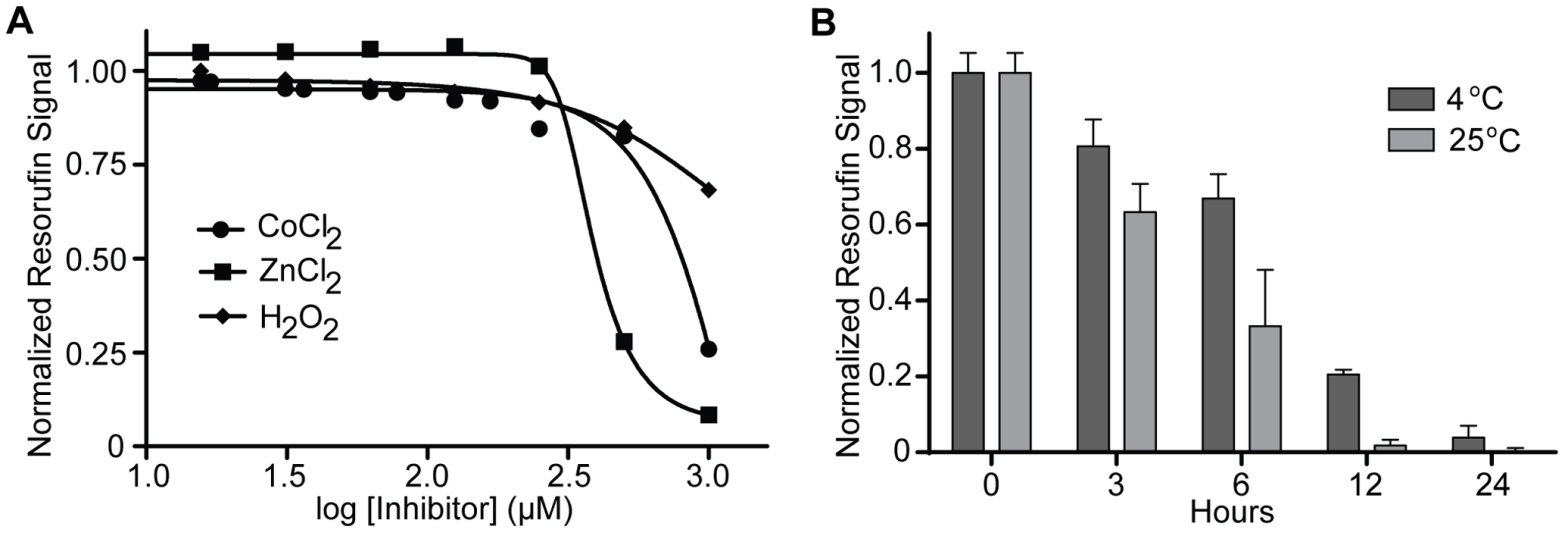
Limitations of the GABase assay. **A.** Inhibitors of the GABase assay. Titrations of potential inhibitors of the GABase assay were carried out and signal was measured after 30 minutes. The assay demonstrated inhibition by the heavy metals cobalt (IC_50_ = 1 mM) and zinc (IC_50_ = 400 µm) at high concentrations. There is weak inhibition by hydrogen peroxide at high sample concentrations (1 mM). Data were obtained in triplicate. **B.** Reaction mix decay kinetics. Following preparation of complete reaction mix, the mix was stored in the dark at either 4°C or 25°C. Following the times listed, the reaction mix was added to a fixed concentration of GABA standard. Signal obtained at different time points was normalized to the signal obtained at time zero. Data were obtained in triplicate.

Several biologically-relevant molecules have the potential to inhibit the assay. Cobalt, commonly used to pharmacologically simulate hypoxia [51], [52] can inhibit diaphorase [53]. Zinc is a potentially relevant metal in neuronal biology as synaptic zinc can regulate neuronal excitability and modulate GABA receptors [54]–[56]. Both metals showed an inhibitory effect on the assay, but only at high sample concentrations, with an approximate IC_50_ of 1000 µM for cobalt and 400 µM for zinc. Oxidative damage from hydrogen peroxide can inhibit NADH-producing enzymes of the tricarboxylic acid (TCA) cycle [57], suggesting that peroxide could inhibit NADH production in the coupled assay. However, only a weak effect of peroxide was observed with approximately 30% inhibition at 1 mM concentration in the sample (**Fig. 8A**).

Given the presence of labile components in the reaction mix, we investigated the stability of the reaction mix at two temperatures, 4°C and room temperature (RT), over a course of 24 hours. The reaction mix was more stable when kept at 4°C compared to RT, but still decayed over time, retaining only 66% of its activity at 6 hours and demonstrating complete loss of activity by 24 hours following preparation (**Fig. 8B**). For this reason, fresh reaction mix was prepared prior to each assay.

### Validation of the Assay

The correlation between *GAD1* mRNA overexpression and decreased disease-free survival in prostate adenocarcinoma and overall survival in clear cell renal cell carcinoma prompted us to examine the potential role of GAD1 in NE metabolism. Although not documented previously in cancer, GAD activity is increased in other organisms such as plants and bacteria in response to biotic and abiotic stressors [58], such as acidity. In addition, evaluation of the GABA shunt pathway demonstrates that glutamate, the substrate for GAD1, may be derived from alpha-ketoglutarate from the TCA cycle or alternatively from glutamine. This suggested that perturbations to the extracellular environment, specifically pH changes and nutrient deprivation, could modulate the concentration of GABA in cancer cells.

The robust concentration of GABA in PNEC cells coupled with previous findings that GABA shunt components, specifically GAD1, are enriched in PNEC cells relative to other prostate adenocarcinoma cells [25] prompted us to use this cell line to investigate environment stress-induced modulation of GABA.

**Figure 9:**
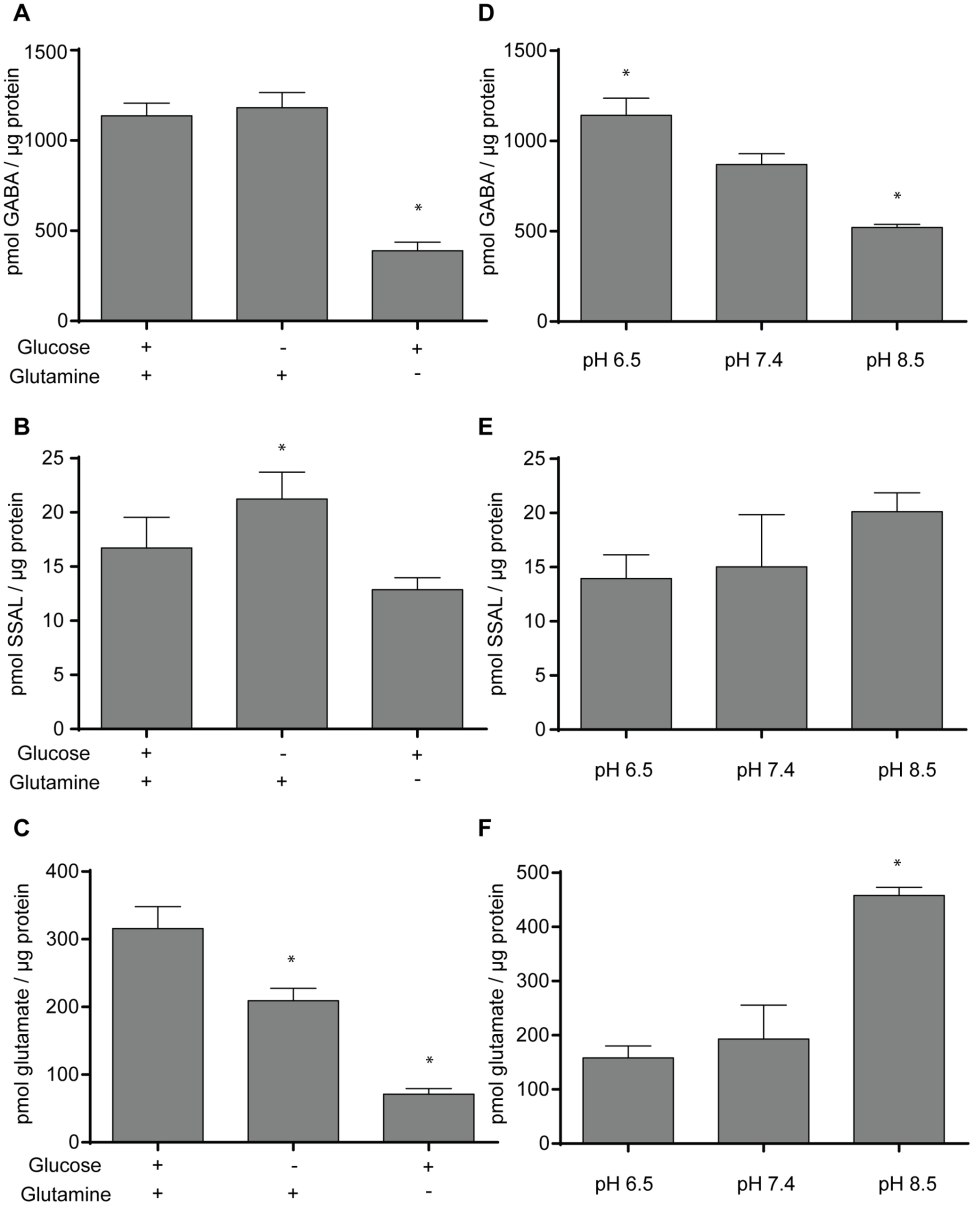
Quantitation of GABA, SSAL, and glutamate in PNEC cells following 24 hours of pH stress and nutrient deprivation. The effects of 24-hour glucose and glutamine deprivation on **A.** GABA, **B.** SSAL, and **C.** glutamate levels in PNEC cells were measured. Nutrient deprivation experiments were performed in substrate-deficient DMEM/F12 media containing dialyzed fetal calf serum supplemented with 6 mM glutamine and/or 17.5 mM glucose for 24 hours. The effects of 24-hour pH stress at pH 6.5 and 8.5 relative to physiologic pH 7.4 on **D.** GABA, **E.** SSAL, and **F.** glutamate levels in PNEC cells were measured. Metabolite levels in the cell lysates was assayed and normalized to total cellular protein. * Significance relative to control (p<0.05). Data represents quadruplicate measurements.

We first quantitated GABA in the PNEC cell line following 24 hours of glucose or glutamine deprivation. Relative to control (i.e., conventional media with dialyzed serum), there was no statistically significant difference in the amount of GABA in glucose-deprived PNEC cells (1137 versus 1182 pmol GABA/µg protein, respectively). However, glutamine deprivation significantly decreased cellular GABA (390 pmol GABA/µg cellular protein) relative to control. Interestingly, levels of SSAL were relatively unaffected by these stresses, with the exception of glucose deprivation. Glucose deprivation induced mildly increased SSAL (21.3 pmol SSAL/µg protein) compared to control (16.7 pmol SSAL/µg protein), just achieving statistical significance (p<0.05) (**Fig. 9**).

Besides quantitating GABA as well as its downstream metabolite, SSAL, under stresses, we also measured the upstream metabolite, glutamate, in PNEC cells under these same stresses. We employed a published resazurin-based enzymatic assay for the detection of glutamate [41] to determine glutamate levels in stressed PNEC cells. Interestingly, relative to control, glutamate levels were significantly decreased by glucose deprivation (316 versus 209 pmol/µg protein, respectively) and most pronounced by glutamine deprivation (71 pmol/µg protein) (**Fig. 9**).

Enzymes within the GABA shunt have different pH optima [26]. For example, mammalian GAD1 has an optimal pH range of 6–7 and mammalian ABAT and ALDH5A1 have an optimal pH range between pH 8–9. This suggested that acidic conditions would favor the synthesis of GABA and, conversely, alkaline conditions would favor the metabolism of GABA. Following 24 hours of exposure to an environmental pH of 6.5, GABA content in PNEC cells was significantly elevated relative to a pH of 7.5 (1142 versus 870 pmol/µg protein). Conversely, GABA content was significantly decreased in PNEC cells exposed to pH 8.5 (521 pmol/µg protein) (**Fig. 9**). Despite these changes in GABA, there were no statistically different changes in levels of SSAL relative to control (**Fig. 9**).

Glutamate levels in PNEC cells under pH stress were also measured. Although there was no significant difference in the amount of glutamate in PNEC cells in pH 6.5 versus pH 7.5 (158 versus 193 pmol/µg protein, respectively), there was significantly elevated glutamate at pH 8.5 (458 pmol/µg protein) (**Fig. 9**).

To explain these changes in PNEC cell metabolites, we investigated enzymatic activity of three key components involved in glutamate, GABA, and SSAL metabolism. In addition to modifying our novel assay to measure GABA produced by GAD activity, we also utilized conventional enzymatic assays for GLUL and ALDH5A1.

**Figure 10:**
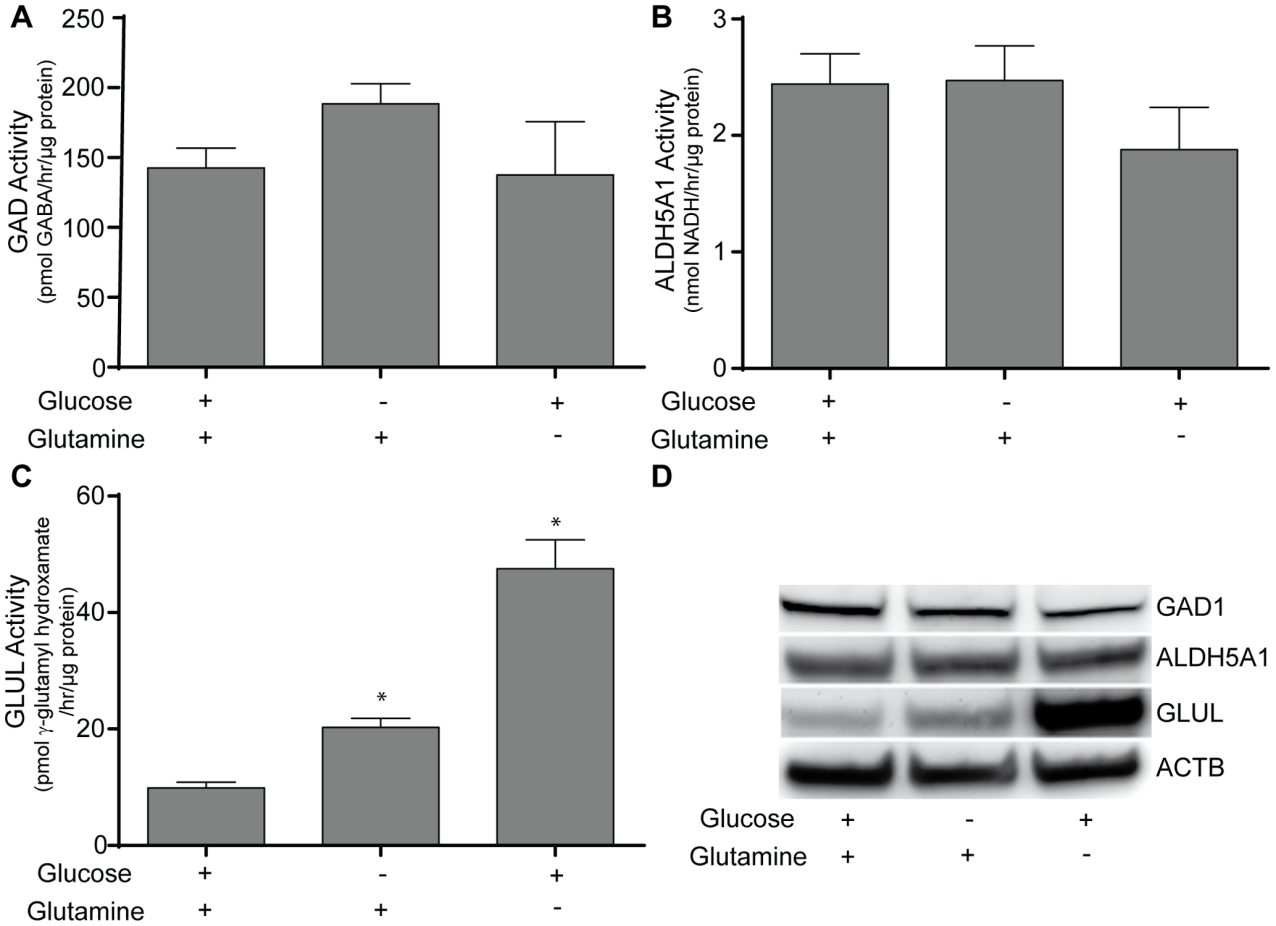
Enzyme activity measurements of GAD1, GLUL, and ALDH5A1 in PNEC cells following nutrient deprivation. The effects of 24-hour glucose and glutamine deprivation on **A.** GAD1, **B.** GLUL, and **C.** ALDH5A1 levels in PNEC cells were measured. **D.** Protein expression of enzymes correlate with enzyme activities. Beta actin (ACTB) is provided as a loading control. Nutrient deprivation experiments were performed as described above. Enzyme activity in the cell lysates was assayed and normalized to reaction time and total cellular protein. * Significance relative to control (p<0.05). Data represents quadruplicate measurements.

There was no significant difference in GAD activity following glucose or glutamine deprivation **(** **Fig. 10** **)**. Of note, there was a trend toward increased GAD activity that could correlate with the significantly increased SSAL levels following glucose deprivation (188.4 versus 142.5 pmol GABA/hr/µg protein). ALDH5A1 activity, similarly, did not demonstrate any significant differences in activity. However, the most notable differences were seen in GLUL activity. Interestingly, glucose deprivation significantly increased GLUL activity (20.27 versus 9.88 pmol γ-glutamyl hydroxamate/hr/µg protein). Glutamine deprivation, as expected, had the most dramatic increase in GLUL activity (47.5 pmol γ-glutamyl hydroxamate/hr/µg protein). We then assessed protein expression levels of these enzymes to correlate with their activities. No definite changes in expression were seen with GAD1 or ALDH5A1. However, GLUL expression was increased in both glucose and glutamine deprivation (**Fig. 10D**).

**Figure 11:**
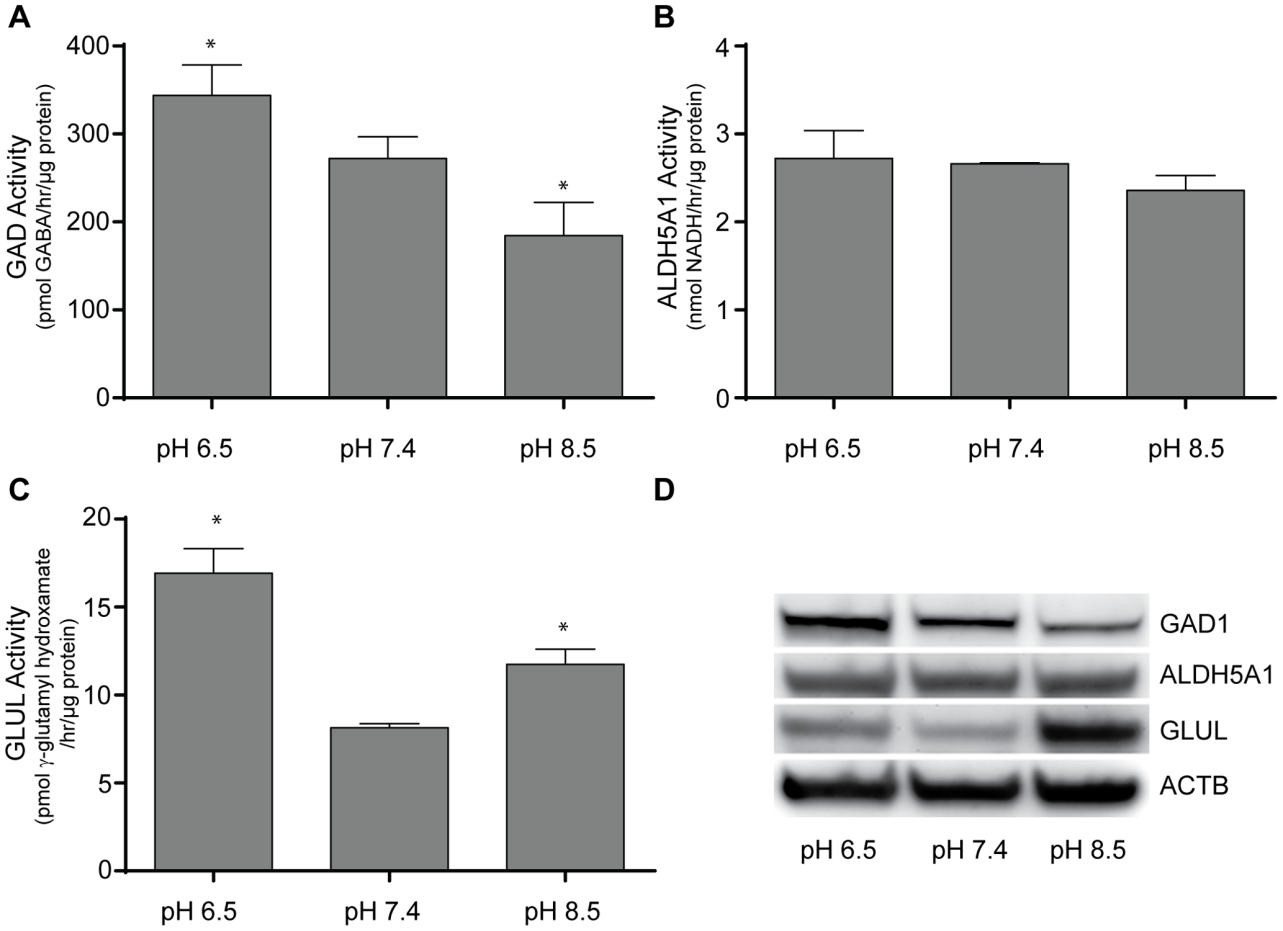
Enzyme activity measurements of GAD1, GLUL, and ALDH5A1 in PNEC cells following pH stress. The effects of 24-hour acid and alkaline stress on **A.** GAD1, **B.** GLUL, and **C.** ALDH5A1 levels in PNEC cells were measured. D. Protein expression of enzymes correlates with enzyme activities. Beta actin (ACTB) is provided as a loading control. Enzyme activity in the cell lysates was assayed and normalized to reaction time and total cellular protein. * Significance relative to control (p<0.05). Data represents quadruplicate measurements.

The effects of pH on GAD activity were more pronounced. Interestingly, acidic pH significantly increased GAD activity (344 versus 272 pmol GABA/hr/µg protein). Conversely, alkaline pH significantly decreased GAD activity relative to physiologic pH (185 pmol GABA/hr/µg protein). The effect of pH on GLUL activity was different, however. Although acidic pH increased GLUL activity (16.93 versus 8.15 pmol γ-glutamyl hydroxamate/hr/µg protein), alkaline pH also increased GLUL activity (11.74 pmol γ-glutamyl hydroxamate/hr/µg protein). The effect of pH on ALDH5A1 activity was different altogether, with statistically-unchanged activity in acidic versus physiologic pH (2.72 versus 2.66 nmol NADH/hr/µg protein) as well as alkaline pH (2.36 nmol NADH/hr/µg protein). Herein, protein expression correlated with enzyme activity. GAD1 expression was increased in acidic pH and decreased in alkaline pH, GLUL expression was increased both in acidic and alkaline pH, and ALDH5A1 was unchanged (**Fig. 11**).

## Discussion

Using previously published datasets characterizing gene expression in prostate and renal cancers [39], [40], a potentially deleterious effect of *GAD1* overexpression and *GLUL* underexpression on patient survival was identified. Both enzymes use glutamate and therefore compete for the same substrate, suggesting the presence of a metabolic antagonism where glutamate conversion to GABA over glutamine is favored in the context of cancers associated with poorer outcomes. Furthermore, the unique overexpression of *GAD1* mRNA in a select cohort of castrate-resistant prostate cancer cell lines was revealed. However, the clinical significance of GAD1 in castrate-resistant prostate cancer remains to be clarified, as the number of GAD1-overexpressing prostate cancers identified in the public databases were small and could not be accurately correlated with castrate-resistant disease. Nonetheless, increased protein expression of the NE enzyme, dopa decarboxylase (DDC; E.C. 4.1.1.28) correlates with castrate-resistant disease [21], further supporting a NE program associated with castrate-resistant prostate cancer. These analyses need to be expanded to larger patient populations and further evaluated with rigorous immunohistochemical and metabolomics methods to further define the prognostic significance of GAD1 and its related pathway components on patient survival and disease recurrence. Our results are further supported by studies documenting the proliferative effects of GABA on cancer cells, the expression of GAD1, and the expression of GABA receptors in multiple cancers throughout the body [59]–[62].

The GABA shunt is conserved in prokaryotes and eukaryotes and is upregulated in the context of multiple stresses. For example, glutamic acid decarboxylase is widely implicated in the survival of prokaryotes and plants in acidic environments [58], [63]–[67]. However, the mechanisms by which glutamic acid decarboxylase allows organisms to survive acidic stress remains unclear. The apparent role of the GABA shunt in the evasion of environmental stresses to promote survival in other organisms is intriguing as such stresses are also significant in the cancer cell microenvironment and may therefore be involved in the ability of cancer cells to survive stress.

Many studies have been conducted on the effects of the cancer microenvironment on tumor physiology. For example, acidity has been demonstrated to drive metastasis [68], while alkalization of the tumor microenvironment with sodium bicarbonate reduces metastasis [69]–[71]. Our findings suggest an interplay between the cancer microenvironment, cancer metabolism, and metastatic potential of cancer cells. It has been documented previously that GAD1-mediated GABA synthesis and signaling through GABA-B receptors facilitate the expression of matrix metalloproteinases and invasion of prostate cancer cells [72]. We suggest that activity of the GABA shunt, at least in prostate cancer cells, may represent a metabolic pathway through which the microenvironment may stimulate metastasis and may also represent a mechanism by which bicarbonate therapy acts to reduce metastasis.

Herein, we demonstrated that alterations in extracellular pH can modulate levels of the GABA shunt metabolites glutamate and GABA through modulation of the expression and activity of the enzymes GAD1 and GLUL. Under acidic conditions, GAD1 protein and activity are increased in PNEC cells correlating with increased GABA concentration. Interestingly, GLUL protein levels and activity are also increased under acidic pH. Despite the increased activity of both of these enzymes involved in glutamate metabolism, there are no statistically significant differences in glutamate concentration under acidic pH. These findings could represent a complex manifestation of metabolic flux through additional metabolic as well as protein synthetic pathways in which glutamate is involved.

The effects of alkalinity on the GABA shunt are equally as intriguing. Exposure to an alkaline pH of 8.5 is met with decreased GAD1 activity as well as a decrease in GABA and an increase in glutamate concentration. Although these changes in metabolite concentration are expected for decreased GAD1 activity, there is also an increase in GLUL activity under alkaline pH. Of note, protein levels of GLUL in alkaline pH were higher compared to acidic pH, despite similar enzyme activity. This could be due to the presence of endogenous enzymatic inhibitors in complex cell lysate or could represent metabolic variability among passages in cell culture, although all cells used in this study were of low culture number. It is currently unclear if the mechanism underlying upregulation of GLUL or its predicted increase in glutamine concentration under alkaline pH is the same as that under acidic pH. The significant increase in glutamate concentration under alkaline, but not acidic pH further underscores the potential for complex metabolic fluxes of glutamate, glutamine, and GABA during pH stress. At this point, we hypothesize that the decrease in GABA under alkaline pH may be related to a combination of substrate competition from increased GLUL as well as decreased GAD1 activity.

The mechanism underlying relatively stable expression of ALDH5A1 under acid and alkaline stress as well as its known regulation by the cellular redox state [73] suggests that the metabolism of GABA into succinate may be more dependent upon post-translational regulation than gene expression. Although the activity of ALDH5A1 is optimal at a pH of 8–9 [26], there was no evidence of significantly different changes in enzyme activity in PNEC cell lysates. Although factors such as extracellular pH, pCO_2_, bicarbonate, and lactate can directly affect intracellular pH [74], [75], it is currently unclear if ALDH5A1 activity is directly modulated by alterations in intracellular pH. In addition, the role of ABAT in GABA metabolism remains to be elucidated. Unchanged concentration of SSAL under pH stress may also represent the effect of complex metabolic fluxes, specifically from carbons through the urea cycle arm of the GABA shunt [13].

The relative contributions of glucose and glutamine to GABA synthesis in cancer cells are incompletely understood. However, general contributions of glucose and glutamine to cancer cell metabolism have been widely documented [76]. Of note, glutamine is an alternative substrate to glucose for the synthesis of nucleotides and lipids and a source for ATP [77], [78]. In addition, the contributions of glutamine metabolism toward cancer cell stress survival have been studied to some extent with evidence that cancer cell survival in hypoxia may be dependent on glutamine and glutamate metabolism [79]. Despite no significant change in GAD1 expression or activity following glutamine deprivation, we identified decreased glutamate and GABA, correlating with increased GLUL activity, as a potential mechanism to increase cellular stores of glutamine. This further supports a potential metabolic antagonism between GLUL and GAD1 that we also identified under alkaline conditions and suggested from the survival analysis. A trend toward decreased SSAL and ALDH5A1 activity following glutamine deprivation suggests decreased flux through the GABA shunt as a result of glutamine deprivation. These findings provide further support for glutamine deprivation as an anticancer therapy to decrease GABA synthesis.

Although glucose deprivation did not result in significant changes in GAD1 expression or activity, there was also a potential trend toward increased GAD1 activity as well as significantly increased SSAL, suggesting that flux through the GABA shunt is maintained or potentially increased during glucose deprivation. Of note, our glucose deprivation experiments were conducted in the absence of exogenous pyruvate, which could potentially act as carbon sources for the TCA cycle. It is possible that the GABA shunt in glucose deprivation may represent a mechanism to produce NADH through the activity of ALDH5A1 and supply carbons to the TCA cycle, presumably through glutamine and potentially the urea cycle, although this remains to be demonstrated with more complex metabolic flux analysis. Increased GLUL activity in glucose deprivation is likely a manifestation of depleted glutamine reserves in cell culture media (used at 6 mM in these experiments) further supporting the need for glutamine as a carbon source for cells in the absence of glucose.

In summary, we have described a new fluorometric assay that has provided a first glimpse into how pH and nutrient stress modulate GABA in prostate NE cancer cells. This presents a novel regulatory mechanism by which cancer cells can respond to environmental stresses. This assay can be widely adapted into single microplate or high throughput formats to measure GABA content in multiple samples under multiple conditions, treatments, or time points.
